# Investigating the Efficacy of an 18-Week Postpartum Rehabilitation and Physical Development Intervention on Occupational Physical Performance and Musculoskeletal Health in UK Servicewomen: Protocol for an Independent Group Study Design

**DOI:** 10.2196/32315

**Published:** 2022-06-01

**Authors:** Kirsty Jayne Elliott-Sale, Emma Louise Bostock, Thea Jackson, Sophie Louise Wardle, Thomas James O'Leary, Julie Patricia Greeves, Craig Sale

**Affiliations:** 1 Musculoskeletal Physiology Research Group Sport, Health and Performance Enhancement Research Centre, School of Science and Technology Nottingham Trent University Nottingham United Kingdom; 2 Institute of Sport Manchester Metropolitan University Manchester United Kingdom; 3 Army Health and Performance Research Army Headquarters Andover United Kingdom

**Keywords:** performance, servicewomen, postnatal, pelvic health, exercise, intervention, musculoskeletal, well-being

## Abstract

**Background:**

Postpartum women are at an increased risk of pelvic floor dysfunction, musculoskeletal injury, and poor psychological health and have reduced physical fitness compared to before pregnancy. There is no formal, evidence-based rehabilitation and physical development program for returning UK servicewomen to work following childbirth.

**Objective:**

This study aims to examine the efficacy of a rehabilitation and physical development intervention for returning postpartum UK servicewomen to occupational fitness.

**Methods:**

Eligible servicewomen will be assigned to a training or control group in a nonrandomized controlled trial 6 weeks after childbirth. Group allocation will be based on the location of standard pregnancy and postpartum care. The control group will receive standard care, with no prescribed intervention. The training group will start an 18-week core and pelvic health rehabilitation program 6 weeks post partum and a 12-week resistance and high-intensity interval training program 12 weeks post partum. All participants will attend 4 testing sessions at 6, 12, 18, and 24 weeks post partum for the assessment of occupational physical performance, pelvic health, psychological well-being, quality of life, and musculoskeletal health outcomes. Occupational physical performance tests will include vertical jump, mid-thigh pull, seated medicine ball throw, and a timed 2-km run. Pelvic health tests will include the Pelvic Organ Prolapse Quantification system, the PERFECT (power, endurance, repetitions, fast, every contraction timed) scheme for pelvic floor strength, musculoskeletal physiotherapy assessment, the Pelvic Floor Distress Inventory–20 questionnaire, and the International Consultation on Incontinence Questionnaire–Vaginal Symptoms. Psychological well-being and quality of life tests will include the World Health Organization Quality of Life questionnaire and the Edinburgh Postnatal Depression Scale. Musculoskeletal health outcomes will include body composition; whole-body areal bone mineral density; tibial volumetric bone mineral density, geometry, and microarchitecture; patella tendon properties; muscle architecture; muscle protein and collagen turnover; and muscle mass and muscle breakdown. Data will be analyzed using linear mixed-effects models, with participants included as random effects, and group and time as fixed effects to assess within- and between-group differences over time.

**Results:**

This study received ethical approval in April 2019 and recruitment started in July 2019. The study was paused in March 2020 owing to the COVID-19 pandemic. Recruitment restarted in May 2021. The results are expected in September 2022.

**Conclusions:**

This study will inform the best practice for the safe and optimal return of postpartum servicewomen to physically and mentally demanding jobs.

**Trial Registration:**

ClinicalTrials.gov NCT04332757; https://clinicaltrials.gov/ct2/show/NCT04332757

**International Registered Report Identifier (IRRID):**

DERR1-10.2196/32315

## Introduction

### Background

Women experience physiological changes during pregnancy and following childbirth, which can lead to pelvic health dysfunction [1-10], musculoskeletal injury [11], increased ligament laxity [12,13], and decreased bone mineral content [14-18]. After childbirth, the pelvic floor can be weakened and sometimes injured, with symptoms of urinary incontinence [1,2,4,7,9], fecal incontinence [1,3,6], and pelvic organ prolapse [1,2,7] commonly reported. Postpartum health problems may negatively influence quality of life and emotional well-being [19]. Postpartum depression is common in the months after childbirth [20-22]; a *m*-analysis of 58 studies reported a prevalence of 17% in mothers [20].

Evidence suggests that exercise is an important consideration for improving maternal health after childbirth [23]; however, few studies exist whereby training interventions seek to address musculoskeletal, physiological, and psychological implications of pregnancy. Previous training interventions in women without pregnancy- or childbirth-related medical pathologies have included home-based pelvic floor exercise programs [24-27], resistance training programs [28], or combined aerobic and resistance training programs [29,30]. Pelvic floor muscle exercise interventions have been predominantly home-based pelvic floor exercises, with limited supervision and consideration of functional rehabilitation of the pelvic floor muscles, which may account for the variability in the success of improving outcomes, including pelvic organ prolapse [24], urinary incontinence [25], vaginal symptoms [24,26], sexual function [26], and diastasis rectus abdominis [27]. Resistance training interventions have resulted in improved muscle strength in postpartum women [28], although a combined intervention of supervised resistance and aerobic training [29,30] may have superior health benefits. No studies have used a combined training intervention including pelvic floor rehabilitation, resistance training, and high-intensity interval training (HIIT) for postpartum women.

Physically arduous occupations often require women to complete physical activities, such as load carriage and heavy lifting. UK servicewomen can return to physically and psychologically demanding roles after 2 weeks of compulsory maternity leave. Previous data show that British Army servicewomen return to work within the first year after childbirth and are at greater risk of illness and injury during this time than before pregnancy [31]. US military servicewomen do not achieve prepregnancy fitness levels at 6 months following childbirth [32-34], and servicewomen commonly experience symptoms of fatigue, depression, and anxiety upon returning to work [33,35].

In the UK Armed Forces, there are differences among services in the approach to returning women to physical training following childbirth. Army servicewomen are provided with a 6-to 12-week return to fitness program before they are (1) permitted to resume normal military duties and (2) required to undergo annual fitness testing. Royal Air Force and Royal Navy servicewomen do not receive a phased return to physical activity, unless specifically requested; an 18-month opt-out of mandatory fitness testing can be requested following childbirth.

### Objectives

The *Interim Report on the Health Risks to Women in Ground Close Combat Roles* calls for strategies to optimize the physical and mental resilience of postpartum servicewomen on return to work to achieve safe and effective integration of women into the military workforce [36]. There is no evidence on how to rehabilitate to full duty following childbirth. This study aims to investigate the efficacy of a postpartum combined rehabilitation (ie, pelvis, hip, and abdominal specific exercises) and physical development (ie, resistance training and HIIT) program on occupational physical performance, musculoskeletal health, pelvic health, psychological well-being, and quality of life. The rehabilitation element of the exercise intervention will target strengthening of the pelvis, hip, and abdominal muscles 6 weeks before commencing the high-intensity aerobic and resistance training.

## Methods

### Study Design and Setting

This study is known as the PERFORM (Postpartum Exercise and Return to Fitness: Optimize Readiness for Military Mums) study (ClinicalTrials.gov NCT04332757). This study will use an independent group design; participants will join either a control or intervention group based on their location of standard pregnancy/postpartum care. This design was chosen to geographically separate groups and reduce the likelihood of the intervention being shared with control participants, who may change their usual behavior. The intervention sites will be selected based on their proximity to the training facilities. The control group will receive standard postpartum care with no formal intervention, whereas the intervention group will receive standard postpartum care plus an 18-week phased rehabilitation and physical development program between 6 and 24 weeks post partum.

**Figure 1:**
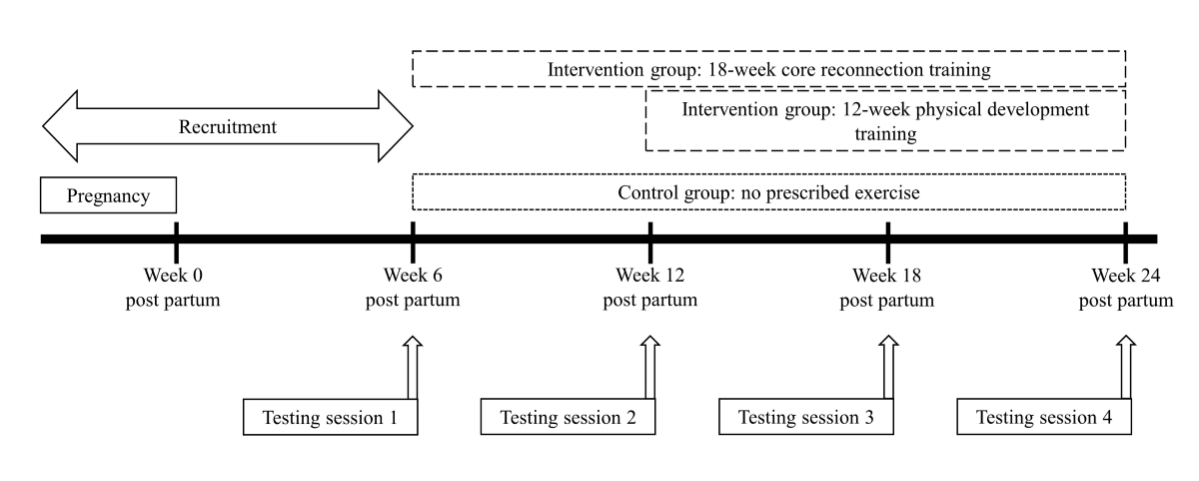
Study schematic.

**Figure 2:**
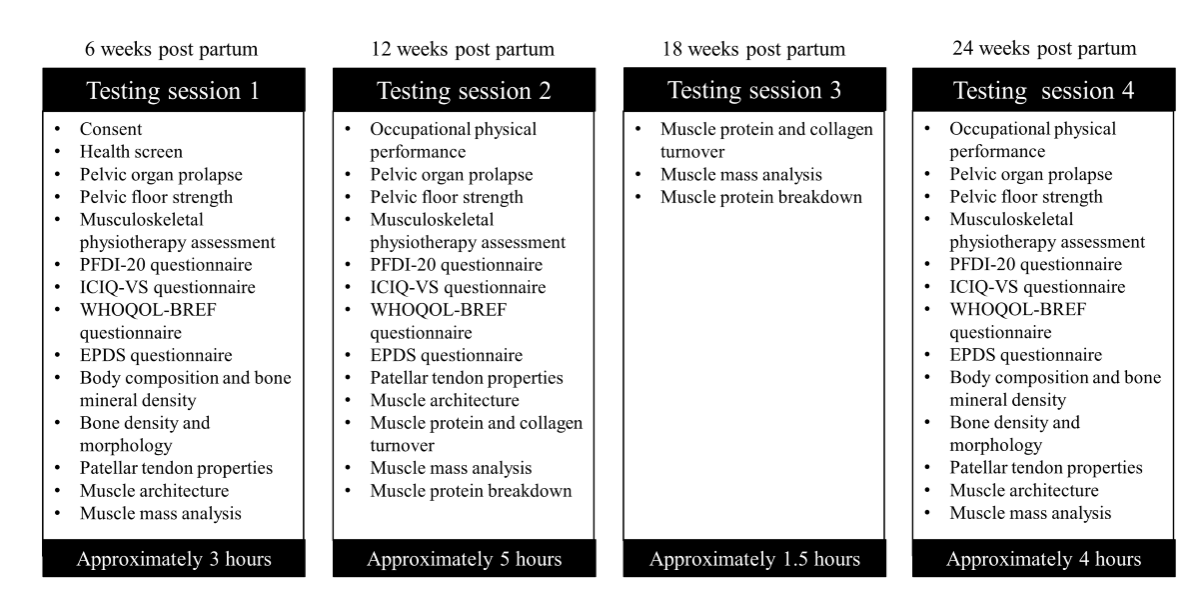
Testing procedures. EPDS: Edinburgh Postnatal Depression Scale; ICIQ-VS: International Consultation on Incontinence Questionnaire–Vaginal Symptoms; PFDI-20: Pelvic Floor Distress Inventory–20; WHOQOL-BREF: World Health Organization Quality of Life.

Participants will attend 4 laboratory-based testing sessions, completed at 6, 12, 18, and 24 weeks post partum (Figure 1). Testing session 1 will be completed as soon as possible after the participants have had their 6-week postpartum general practitioner check. Measurements being completed at each testing session are summarized in Figure 2.

### Recruitment Strategies

Participants will be recruited through leaflets provided by their midwife or through posters and leaflets displayed around the targeted garrisons. Recruitment information will be included in routine information downloads to staff at chosen units, and the study will be promoted through internal social media channels. Recruitment will be performed on an opt-in basis, wherein the participants contact the research team.

### Participants and Eligibility Criteria

Participants will be healthy servicewomen aged ≥18 years, recruited in the antenatal and postpartum periods, before 6 weeks post partum. Participants will have completed their 6-week postpartum checkup with their general practitioner and will have been cleared to exercise. Participants will be excluded if they have been diagnosed with postnatal depression or other mental health conditions that require specialist psychiatric secondary care. Participants will complete a health screening questionnaire when attending their first testing session to confirm the absence of any condition or injury that may affect their ability to exercise. On their first testing session, participants must be free from gynecological-based contraindications to exercise, as assessed by the study’s specialist pelvic health physiotherapist. During each testing session, participants will be asked to declare their menstrual, contraceptive, and breastfeeding status. Participants must consent to a pregnancy test before scans at 6 and 24 weeks post partum.

### Intervention

#### Overview

The control group will receive standard postpartum care only, which consists of a 6-week postpartum check at a civilian general practice or a Defence primary health care facility and referral to (1) secondary care pelvic health physiotherapy if women present with symptoms of urinary incontinence and (2) musculoskeletal physiotherapy if they present with *rectus diastasis* or a musculoskeletal injury. Control participants will not be prescribed exercises but will not be prevented from choosing to exercise during the study period. A log of all exercise activities will be collected from control participants. The intervention group will receive standard postpartum care and complete a phased 18-week combined rehabilitation and physical development program. The rehabilitation element of the program was designed for this study by specialist pelvic health physiotherapists. The program, known collectively as *core reconnection training*, targets pelvic floor function and pelvic, hip, and abdominal strength. The *physical development training* is an adapted version of the SPARTA (Soldier Performance and Readiness as Tactical Athletes) training program [37] and consists of resistance training and HIIT.

#### Core Reconnection Training

Following testing session 1, participants in the intervention group will commence 18 weeks of home-based core reconnection training from weeks 6 to 24 post partum (Table 1). Participants will complete 3 core reconnection training sessions per week, each lasting approximately 30 minutes. Each session will include 6 exercises progressing in difficulty throughout the intervention. Participants will complete 3 to 4 sets of 8 to 12 repetitions of each exercise. Participants will be advised to reduce the number of repetitions of more challenging exercises (eg, press-ups) to 3 to 4 sets of 5 repetitions. Exercises will be modifiable depending on individual ability and symptoms. Participants will be advised to be aware of symptoms of incontinence, a heaviness or dragging sensation in the pelvic area (ie, indicator of prolapse), a pendular abdomen or noticeable gap in the midline of the abdominal wall (ie, indicator of *rectus diastasis*), and pelvic or lower back pain. If any of these symptoms are experienced, participants will be asked to contact the study’s pelvic health physiotherapist before continuing with training. Participants will be given written instructions with images of each exercise and will be provided with access to all exercises in video form with verbal instructions. Core reconnection training sessions will not be routinely supervised.

**Table 1:** Core reconnection training.

	Exercise 1	Exercise 2	Exercise 3	Exercise 4	Exercise 5	Exercise 6
Week 1	Arm circles + heel slide	Glute bridge + squeeze	Side kick	Bent knee fall out	Toe slide all fours	Tempo squat to chair
Week 2	Toe taps + arms	Glute bridge + band pull	Side kick straight leg	Eccentric curl down	Toe slide + arm slide	Tempo squat to chair
Week 3	Arm circles + heel slide in tabletop	Single-leg glute bridge	Sidestep + band	Abdominal prep	Superman	Squat to tip toes
Week 4	Running man	Monster walk + band	Mountain climber	Eccentric curl down + rotation	Superman knee to elbow	Static lunge + arms overhead
Week 5	Knee pull	Banded deadlift	Mountain climber knee to elbow	Oblique prep	All four hover	Lunge + rotation band pull
Week 6	Single-leg squat + rotation	Banded deadlift	Mountain climber (increased pace)	Russian twist	Bear crawl	Lunge + rotation band pull
Week 7	Opposite knee to elbow in standing	Single-leg deadlift	Press-up to chair	Side-lying double-leg lifts	Plank to pike	Lateral lunge
Week 8	Dead bug	Single-leg deadlift	Press-up to chair	Side-lying lift legs + hip abduction	Plank to pike + opposite toe touch	Lateral lunge + weight
Week 9	Dead bug	Single-leg deadlift + weight	Walkout to plank wide stance	Side plank bent knees (on elbow)	Plank from knees	Lateral lunge + weight
Week 10	Dead bug + curl up	Single-leg deadlift + weight	Walkout narrow stance	Side plank bent knee + rotation	Prone pull with hip extension	Hip thrust on sofa
Week 11	Dead bug + curl up	Running man	Walkout chest to floor	Side plank straight leg + rotation	Plank holds on chair	Hip thrust
Week 12	Dead bug + hand weights	Running man	Walkout chest to floor	Side plank knee to elbow	Plank holds on chair	Hip thrust + weight
Week 13	Opposite knee to elbow supine	Single-leg deadlift + weight	Press-up	Side plank knee to elbow	Plank + shoulder taps	Crab raises
Week 14	Bicycle crunch + knee extension	Single-leg deadlift + weight	Press-up	Side plank + clam	Plank with hip abduction	Crab raises
Week 15	Russian twist legs raised	Hydrant	Press-up + single-arm row	Side plank + clam + sofa or step	Plank to half inch worm	Bridge on sofa or step
Week 16	Russian twist legs raised	Hydrant + hip extension	Press-up + single-arm row	30-second side plank	30-second plank hold	Bridge on sofa or step
Week 17	Bicycle crunch + knee extension	Hydrant + hip extension	Press-up	30-second side plank	Plank to half inchworm	Crab raises
Week 18	Bicycle crunch + knee extension	Hydrant	Press-up	Side plank pulses	30-second plank hold	Crab raises

#### Physical Development Training

##### Overview

**Figure 3:**
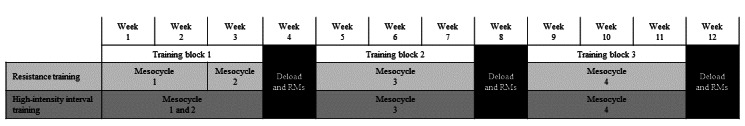
Physical development training mesocycles and training blocks. RM: repetition maximum.

Following testing session 2, the intervention group will undergo a supervised 12-week combined resistance and HIIT program (collectively known as physical development training) from 12 to 24 weeks post partum. Participants will attend 3 supervised sessions per week in a designated gym space. Resistance training and HIIT sections of the program will consist of three 4-week training blocks, with the first 3 weeks of each block for training and the final week of each block for *deload* and assessing 1 repetition maximum (RM; Figure 3). The program incorporates training periods (known as mesocycles), with specific training adaptations at the focus of each mesocycle. The physical development training aims to improve athletic and task-specific capabilities while minimizing the risk of injury. The program is designed to induce physiological adaptation and promote recovery to enhance performance while avoiding overtraining. A warm-up will be completed before each training session, including 12 of the exercises listed in Table 2.

**Table 2:** Physical development training warm-up exercises.

Exercise			Repetitions		Sets, n
**On hands and knees or supine**					
	Fire hydrants	10 each side		1	
	Thread the needle	10 each side		1	
	Lumbar rotations (knee rolls)	10 each side		1	
	T-spine mobility rotations	10 each side		1	
**Standing**					
	Single-leg banded sprinter kickback	10 each side		1	
	Single-leg lateral band quarter squat	10 each side		1	
	Bent over Ys, Ts, and Ws	10 each side		1	
	External shoulder rotations (banded)	10		1	
	Running man	10 each side		1	
	Air squat	10		1	
	Single-leg banded adductor squeeze	10 each side		1	
**Walking**					
	Quad stretch and reach	9 m		1	
	Step and scoop	9 m		1	
	Leg cradle to lateral lunge	9 m		1	
	Toy soldiers	9 m		1	
	Grapevines	9 m		1	
	Bounds (single leg)	9 m		1	
	Bum kicks	9 m		1	
	High knees (walk or run)	9 m		1	

##### Resistance Training Program

Every resistance training session will include five exercises: an upper-body pull, an upper-body push, a lower-body pull, a lower-body push, and a core-focused exercise (Table 3). Only mesocycle 1, day 2, will have 4 exercises, and mesocycle 4, day 3, will have 6 exercises in total. Each of the 3 training days across all mesocycles will have a different focus: day 1 will focus on power, day 2 on strength, and day 3 on stability. Table 4 summarizes the goals of the resistance training blocks. The first mesocycle is designed to prepare the individual with emphasis on form and volume. Mesocycle 2 will include the same movements but under a greater load. Mesocycle 3 will aim to develop greater force production. Mesocycle 4 will serve as the peaking phase for the end points of this study, and the exercises used will be task-specific for active duty.

**Table 3:** Physical development training: resistance training.

Exercise		Sets, n	Repetitions	Rest (minutes)
**Mesocycle 1, day 1**				
	Step up on to box	10	3	0.5
	Mid-thigh clean pull	10	3	0.5
	Medicine ball throw	10	3	0.5
	Lawnmower row	10	3	0.5
	All four hovers	3	30 seconds	1
**Mesocycle 1, day 2**				
	Box squat (below parallel)	10	3	1
	Incline chest press	10	3	1
	Deadlift	10	3	1
	Modified pull-ups	10	3	1
**Mesocycle 1, day 3**				
	Single KB^a^ front squat	3	12	1
	SA^b^ KB neutral press	3	12	1
	SL^c^ deadlift	3	12	1
	All fours SA row	3	12	1
	SA farmer’s walk	3	30 meters	1
**Mesocycle 2, day 1**				
	Step up on to box	4	5	1
	Mid-thigh clean pull	4	5	1
	Medicine ball throw	4	5	1
	Lawnmower row	4	5	1
	All four hovers	3	30 seconds	1
**Mesocycle 2, day 2**				
	Box squat (below parallel)	4	5	1
	Incline chest press	4	5	1
	Deadlift	4	5	1
	Modified pull-ups	4	5	1
	Pallof press	3	10	1
**Mesocycle 2, day 3**				
	Single KB front squat	3	12	1
	SA KB neutral press	3	12	1
	SL deadlift	3	12	1
	All fours SA row	3	12	1
	SA farmer’s walk	4	30 meters	1
**Mesocycle 3, day 1**				
	Depth drop jump	3	3	2
	Power shrug (from floor)	3	3	2
	Chest press on bench	3	5	2
	Bent over row	3	5	2
	Mountain climbers	4	10 seconds	2
**Mesocycle 3, day 2**				
	Back squat	3	3	2
	Close grip chest press	3	3	2
	Deadlift	3	3	2
	Barbell bicep curl	3	3	2
	Bear crawls (forward and back)	3	30 seconds	2
**Mesocycle 3, day 3**				
	Front squat	10	3	1
	Standing military press	10	3	1
	Bent over row	10	4	1
	Bent over lateral raise	3	10	1
	KB swings	3	10	1
**Mesocycle 4, day 1**				
	Depth jump to broad jump	3	3	2
	Modified pull-ups	3	3	2
	Mid-thigh high pull	3	5	2
	SA KB press	3	5	2
	Plate push	4	20 seconds	2
**Mesocycle 4, day 2**				
	Zercher squat	3	2	2
	Incline chest press	3	2	2
	Sumo deadlift	3	2	2
	Inverse row (wide grip)	3	4	2
	Woodchopper circuit	3	5	2
**Mesocycle 4, day 3**				
	Lift and carry task	3	10	1
	Burpee with DB^d^ press	3	8	1
	Sandbag lunge	3	20	1
	Bent over alternate row	3	20	1
	Turkish get-up (first phase)	3	10	1
	Zercher farmer’s walk	3	18 m	1

^a^KB: kettlebell.

^b^SA: single arm.

^c^SL: single leg.

^d^DB: dumbbell.

**Table 4:** Summary of resistance training blocks.

Training block	Mesocycle	Weeks	Mesocycle goal	Example sets and repetitions
Training block 1	1	1-2	General physical preparedness	High number of sets and low number of repetitions (eg, 10 sets of 3 repetitions)
Training block 1	2	3	Preparation for peak force	Lower number of sets and more repetitions (eg, 4 sets of 5 repetitions)
Training block 2	3	5-7	Peak force development	Low number of sets and repetitions (eg, 3 sets of 3 repetitions)
Training block 3	4	9-11	Developing anaerobic power capacity	Low number of sets and repetitions (eg, 3 sets of 3 repetitions); more complex and task-specific exercises for active duty

RM lifts will be completed for the squat, bench press, deadlift, and military press in week 1 of the resistance training program. The deload and RM testing weeks (Figure 3) will be used to reassess RM lifts and ensure recovery between mesocycles and the final testing session. Participants will complete a 1, 2, 3, 4, or 5 RM to avoid overlifting and injury. If a participant completes a 2, 3, 4, or 5 RM, the third column of Table 5 will be used to approximate 1 RM. The 1-RM value will be used to prescribe the training loads for weeks 1, 2, and 3 of each mesocycle. For mesocycle 1, the following steps will be performed:

1. The 1-RM value will be used to calculate an *adjusted RM* at 75% of the 1-RM value.
2. The proportion will be either 85%, 90%, or 95% of the *adjusted RM* and will be used to calculate the training loads for weeks 1, 2, and 3 of the mesocycle.

For mesocycles 2, 3, and 4, the following steps will be performed:

1. The 1-RM value will be used to calculate an *adjusted RM*. This will be based on the number of repetitions they are expected to lift. For example, if they are expected to perform five repetitions, the 5-RM equivalent is calculated using Table 5 and is used as the *adjusted RM*.
2. The proportion will be either 85%, 90%, or 95% of the *adjusted RM* and will be used to calculate the training loads for weeks 1, 2, and 3 of the mesocycle.

For all other weight-related exercises (ie, not squat, bench press, deadlift, and military press), researchers will make a judgment call for the starting weights and will aim to progress the weight lifted across each mesocycle. If participants reach maximum effort during the mesocycle, the weight will be lowered. To monitor the effort during training, a 1 to 10 rating of perceived exertion (RPE) scale will be used. Participants will aim to achieve a score of 6 to 7, 7 to 8, and 9 during weeks 1, 2, and 3 of the mesocycle, respectively. Priority will be given to the correct volume of training, rather than to the correct effort during training, to reduce the risk of injury associated with repetitive maximum efforts. Although the weights to be lifted will be predetermined, there will still be an element of coaching and modification.

**Table 5:** Conversion table for repetition maximum values.^a^

Number of repetitions performed	Percentage of 1 repetition maximum	Multiply weight lifted by:
1	100	1.00
2	95	1.05
3	93	1.08
4	90	1.11
5	87	1.15
6	85	1.18
7	83	1.20
8	80	1.25
9	77	1.30
10	75	1.33
11	70	1.43
12	67	1.49
15	65	1.54

^a^Adapted from the study by Haff and Triplett [38].

##### HIIT Program

HIIT exercises (Table 6) will be completed in the same session as the resistance training program. Exercises on each day of the HIIT program will target a different outcome (Table 7). A polar heart rate (HR) monitor will be worn during sessions to monitor the HR and ensure that the participants are exercising at the prescribed intensity (Polar H7 Bluetooth 4.0 and Polar Beat app, version 3.4.4). The target HR will be calculated as a percentage of the maximum HR based on the following equation: 220 − age. The average HR and peak HR will be recorded after each HIIT session. A 1 to 10 RPE scale will be used to monitor perceived exertion during the HIIT exercises. The HR will be monitored throughout the HIIT exercises and will be used alongside RPE to achieve the target HR as closely as possible throughout the sessions.

**Table 6:** Physical development training: high-intensity interval training.

Mesocycle, day, and exercise				Sets, n		Work, seconds		Rest, seconds
**Mesocycle 1 and 2**								
	**Day 1**							
		Bike	4		120		30	
		Sandbag lift and carry	2		240		60	
		Reverse lunges alternate legs	7		20		30	
	**Day 2**							
		Rope slams (slow cadence)	10		30		30	
		Farmer’s walk (speed walk)	2		240		60	
		Kettlebell high pulls	7		20		30	
	**Day 3**							
		Bike	3		180		60	
		Side to side step ups to box	10		30		30	
		Triceps dips	7		20		30	
**Mesocycle 3**								
	**Day 1**							
		Bike with resistance	5		90		30	
		Sandbag shoulder to shoulder	10		30		20	
		Ladders forward and backward	5		20		60	
	**Day 2**							
		Rope slams	5		20		50	
		Sandbag carry walk	3		150		60	
		Suspension trainer rows	10		30		30	
	**Day 3**							
		Run	2		240		120	
		Push-ups (Suspension trainer)	10		30		30	
		Side-to-side jumps	5		20		60	
**Mesocycle 4**								
	**Day 1**							
		Sprint (20 m)	10		N/A^a^		30	
		Farmer’s carries	15		5		30	
		Skater jumps	10		30		30	
	**Day 2**							
		Weighted vest jog	3		60		120	
		Ropes (drumming)	9		20		45	
		Suspension trainer rows (overhand grip)	10		30		30	
	**Day 3**							
		Jog	1		480		120	
		Push ups	10		30		30	
		Scissor jumps	10		10		45	

^a^N/A: not applicable.

**Table 7:** Summary of high-intensity interval training blocks.

Mesocycle, day, and goal for the session				Target HR^a^ (% maximum HR)
**Mesocycle 1 and 2**				
	**Day 1: aerobic power**			
		Week 1	75	
		Week 2	80	
		Week 3	85	
	**Day 2: upper-body localized anaerobic power**			
		Week 1	75	
		Week 2	80	
		Week 3	85	
	**Day 3: cardiac output**			
		Week 1	65	
		Week 2	70	
		Week 3	75	
**Mesocycle 3**				
	**Day 1: lower-body** **anaerobic power**			
		Week 1	80	
		Week 2	85	
		Week 3	90	
	**Day 2: upper-body localized anaerobic power**			
		Week 1	80	
		Week 2	85	
		Week 3	90	
	**Day 3: aerobic maintenance**			
		Week 1	65	
		Week 2	70	
		Week 3	75	
**Mesocycle 4**				
	**Day 1: lower-body anaerobic power**			
		Week 1	85	
		Week 2	90	
		Week 3	95	
	**Day 2: upper-body localized anaerobic power**			
		Week 1	80	
		Week 2	85	
		Week 3	90	
	**Day 3: aerobic maintenance**			
		Week 1	65	
		Week 2	70	
		Week 3	75	

^a^HR: heart rate.

#### Exercise Logs

Participants in both groups will be provided with a log to record the exercises performed outside of the supervised study training. Participants will record RPE, exercise type, duration, setting (eg, home or gym), date, and how the session felt. Exercise will be described to participants as a specific form of physical activity that is planned and structured and includes intentional movements intended to improve or maintain physical fitness. An RPE scale will be provided, and participants will select a number between 1 and 10 to express their RPE, ranging from very light (1-2) to maximum effort (9-10). The control group will record all the exercises performed during the 18-week study period. The intervention group will record the core reconnection training sessions as a metric of compliance and any other exercise in addition to the physical development training sessions performed during the study period.

#### Intervention Compliance (Intervention Group Only)

Compliance with weekly core reconnection training will be checked verbally and by using exercise logs. A participant who misses >25% (>3/12) of sessions over 2 consecutive weeks or who fails to achieve at least two of the three supervised physical development training sessions over 2 consecutive weeks, will be approached by one of the research team members to understand the reason for low compliance. These participants will be given an opportunity to increase their participation rate over the following week, if appropriate. Failure to increase participation during this week will result in a participant being considered noncompliant. Before recruitment, participants will be made aware of noncompliance thresholds. Cases of noncompliance will be reported to a trial committee that will attempt to contact the participant before deciding to exclude the individual from further participation in the trial.

### Primary Outcome Measures

#### Occupational Physical Performance Overview

Occupational physical performance will be measured at weeks 12 and 24 using the Role Fitness Test (Entry), which is used by the British Army as a selection standard to confirm the appropriate level of fitness to commence basic training. It involves an isometric mid-thigh pull, seated medicine ball throw, and a timed 2-km run. A vertical jump test will be completed as a military-relevant field-based measure of power. The occupational physical performance tests will be conducted using military equipment and following military procedures (field-expedient measures). As part of military physical performance testing, there is a minimum standard that individuals must achieve, and maximum scores or values are used across these tests. The maximum values will be reported for the occupational physical performance tests in this study.

#### Occupational Physical Performance Tests

##### Isometric Mid-Thigh Pull

An isometric mid-thigh pull will be used to measure the lower-body maximal strength. The isometric mid-thigh pull test will be conducted using a military mid-thigh pull rig, and the test will be conducted in line with military procedures. The bar will be fixed at the height of the participants’ mid-thigh while in slight knee and ankle flexion. Participants will step onto the force platform with their thighs touching the bar, shoulders back, and chest up and them looking straight ahead. After stepping onto the platform, participants will be required to stand still for 1 to 2 seconds while their body mass is recorded, and the system is zeroed. Participants will then adopt the pull position and remove the slack from the bar. They will be instructed, on a count of 3, to pull as hard and fast as possible for 5 seconds. Participants will be familiarized with the technique and given 2 practice attempts before completing a maximal pull. Each participant will complete 3 maximal pulls against the fixed bar with a maximal effort for 5 seconds, with a 2-minute rest between attempts. After each attempt, the maximum score taken from the mid-thigh pull display screen will be recorded, and the best of the three attempts will be reported.

##### Seated Medicine Ball Throw

A seated medicine ball throw will be used to assess upper-body power. Participants will sit with their backs pressed against a wall and legs flat to the floor straight out in front of them. The participants will be instructed to push a 4-kg ball from their chest upward and outward at a 45° angle to try and achieve a maximum distance thrown. Participants will be familiarized with the technique and angle of release before testing. The participants will throw the ball once, and the distance achieved will be recorded. All participants will use the same 4-kg ball when performing this test.

##### 2-km Best-Effort Run

A 2-km best-effort run will be used to assess aerobic fitness. Participants will complete 5 laps of a 400-m running track as quickly as they feel comfortable to do so, and the time will be recorded.

##### Vertical Jump

A vertical jump will be used to assess lower-body power. Participants will complete three maximal vertical jumps using a Takei Vertical Jump Meter (Takei Scientific Instrument Co) to determine jump height and estimate leg power. Participants will be instructed to jump as high as possible while keeping their hands on their hips. There will be a 2-minute rest period between attempts, and the best score of the 3 jumps will be recorded. Participants will be allowed time to familiarize themselves with the procedure before starting the test jumps.

### Secondary Outcome Measures

#### Pelvic Organ Prolapse

Pelvic organ prolapse will be measured by a pelvic health physiotherapist at weeks 6, 12, and 24. Vaginal examination will assess for prolapse using the Pelvic Organ Prolapse Quantification system (POP-Q) [39,40], quantifying pelvic support in stages from 0 to 4, with 0 indicating no prolapse demonstrated and 4 demonstrating full procidentia. A total of 6 defined points (Aa, Ba, C, D, Ap, and Bp) and 3 landmarks (genital hiatus, total vaginal length, and perineal body) will be measured using a *POP-Stix* (POPstix) tool. The hymen will be the fixed point of reference throughout the completion of the POP-Q. Each measurement will be taken in centimeters above or proximal to the hymen (positive number), with the plane of the hymen defined as 0 (Table 8) [39]. All measurements, except for total vaginal length, will be performed with maximal Valsalva. The POP-Q staging criteria are listed in Table 9 [39].

**Table 8:** Description of the 9 measures of the Pelvic Organ Prolapse Quantification system.^a^

Points	Description	Range of values
Aa	Anterior vaginal wall 3 cm proximal to the hymen	−3 to +3 cm
Ba	Most distal position of the remaining upper anterior vaginal wall	−3 cm to +tvl
C	Most distal edge of cervix or vaginal cuff scar	N/A^b^
D	Posterior fornix	N/A
Ap	Posterior vaginal wall 3 cm proximal to the hymen	−3 to +3 cm
Bp	Most distal position of the remaining upper posterior vaginal wall	−3 cm to +tvl
gh^c^	Measured from middle of external urethral meatus to posterior midline hymen	N/A
pb^d^	Measured from posterior margin of gh to middle of anal opening	N/A
tvl^e^	Depth of vagina when point D or C is reduced to normal position	N/A

^a^Adapted from Bump et al [39].

^b^N/A: not applicable.

^c^gh: genital hiatus.

^d^pb: perineal body.

^e^tvl: total vaginal length.

**Table 9:** Pelvic Organ Prolapse Quantification system staging criteria.^a^

Stage	Description
0	Aa, Ap, Ba, Bp=−3 cm and C or D≤−(tvl^b^ − 2) cm
1	Stage 0 criteria not met and leading edge <−1 cm
2	Leading edge≥−1 cm but ≤+1 cm
3	Leading edge>+1 cm but <+(tvl − 2) cm
4	Leading edge≥+(tvl − 2) cm

^a^Adapted from Bump et al [39].

^b^tvl: total vaginal length.

#### Pelvic Floor Strength

Pelvic floor strength will be measured by a pelvic health physiotherapist at weeks 6, 12, and 24 using the PERFECT (power, endurance, repetitions, fast, every contraction timed) scheme [41]. The participant will perform maximum-effort pelvic floor contraction following instructions and a practice attempt. Power will be recorded on a modified Oxford scale of 0 to 5. Endurance will be assessed by counting the number of seconds until maximum voluntary contraction can no longer be sustained and fatigue is reached. Repetitions of this sustained contraction will be performed with a 4-second rest between each contraction. Fast contractions will be performed by 1-second maximum voluntary pelvic floor contraction and 1-second relaxation until a maximum voluntary contraction can no longer be achieved. Overactivity, coordination, and relaxation of the pelvic floor muscle contraction will also be assessed during the digital vaginal examination.

#### Musculoskeletal Physiotherapy Assessment

Musculoskeletal physiotherapy assessments will be performed by a pelvic health physiotherapist at weeks 6, 12, and 24. *Rectus diastasis* will be assessed by measuring the distance between the internal borders of the *m. rectus abdominis*; fingers will be placed vertically on the *linea alba* 4 cm above and below the umbilicus and at the umbilicus during a curl-up movement. Scoring is an approximation of the width separation and the tone and tension of the *linea alba* structure. The larger the score (cm), the greater the separation of the *m. rectus abdominis*.

At weeks 6, 12, and 24, the pelvic health physiotherapist will observe all participants’ posture, movement patterns, and breathing. If the pelvic health physiotherapist deems participants’ movement or breathing patterns to be dysfunctional, exercises included in the core reconnection and physical development training may be modified slightly or additional advice and guidance provided to ensure that the desired outcome of the exercise is achieved.

Load and impact management tests will be performed during testing session 2, before occupational physical performance tests. These tests will be used to establish the participants’ capacity for safe return to running and further participation in the occupational physical fitness tests [42]. Participants will be asked to perform single-leg balance on each leg (10 seconds), 10 single squats on each leg, a jog on the spot for 1 minute, 10 forward bounds, 10 hops in place on each leg, and 10 single-leg running man motions on each leg. After each functional test, participants will be asked to report any symptoms of pain, heaviness or dragging in the vagina, or incontinence.

#### Pelvic Floor Distress Inventory Questionnaire

The Pelvic Floor Distress Inventory–20 (PFDI-20) [43] will be completed at weeks 6, 12, and 24. The PFDI-20 includes 20 questions and 3 scales. The 3 scales are the Pelvic Organ Prolapse Distress Inventory (6 questions), Colorectal-Anal Distress Inventory (8 questions), and Urinary Distress Inventory (6 questions). Participants will record their answers reflecting on the last 6 weeks and use the scale of 0 to 4 to indicate how bothersome the symptoms are. Each scale is scored from 0 (least distress) to 100 (greatest distress). A scale score will be given according to the mean value of all answered items, with the corresponding scale (possible value 0-4) multiplied by 25 (range 0-100). The sum of the scores from the 3 scales combined, ranging from 0 to 300, provides an overall summary score of the PFDI-20.

#### International Consultation on Incontinence Questionnaire–Vaginal Symptoms

The International Consultation on Incontinence Questionnaire–Vaginal Symptoms [44] will be completed at weeks 6, 12, and 24 and uses 3 subscales to evaluate vaginal symptoms (0-53), associated sexual matters (0-58) and impact on quality of life (0-10) over the previous 4 weeks. There are 14 questions for which participants will record the severity of each symptom. Each item includes a bother scale, where 0 represents the least bothersome in each subscale. The bother scales are not incorporated in the overall score but indicate the impact of individual symptoms.

#### World Health Organization Quality of Life Questionnaire

The World Health Organization Quality of Life-BREF questionnaire [45] will be completed at weeks 6, 12, and 24. Participants will respond to the 26 questions reflecting the last 2 weeks. The World Health Organization Quality of Life-BREF questionnaire provides an overall quality of life and well-being score and allows the calculation of four domain scores: physical health, psychological health, social relationships, and environment.

#### Edinburgh Postnatal Depression Scale

The Edinburgh Postnatal Depression Scale will be completed at weeks 6, 12, and 24. The Edinburgh Postnatal Depression Scale contains 10 questions reflecting the past 7 days [46]. The questionnaire will produce a total score of postnatal depression symptoms (range 0-30) with a score of ≥13, indicating a high likelihood of depressive illness. Participants will be informed that they can omit any questions that they do not wish to answer. However, if >20% of the data are missing for an individual, their data will not be included in the subsequent analysis.

#### Body Composition and Bone Mineral Density

Body composition (whole-body fat mass and fat-free mass) and whole-body areal bone mineral density will be measured at weeks 6 and 24 using a whole-body dual-energy x-ray absorptiometry scan (Lunar iDXA, GE Healthcare). Participants will be scanned in minimal clothing and instructed to lie as still as possible for the approximately 7-minute scan. Participants will arrive at the laboratory in a rested state, at least 3 hours in the postprandial state, and euhydrated, having consumed 500 mL of water in the 2 hours before scanning [47].

#### Bone Density and Morphology

A 3D high-resolution peripheral quantitative computed tomography system (XtremeCT II, Scanco Medical AG) will be used to assess volumetric bone mineral density (vBMD), geometry, microarchitecture, and estimated mechanical strength of the nondominant (self-reported) tibia at 6 and 24 weeks post partum. A 3D representation of approximately 10 mm of the tibia in the axial direction, at both the metaphyseal (4% site) and diaphyseal (30% site) tibia, will be obtained from 165 computed tomography slices with an isotropic voxel size of 61 μm. Tibial length will be measured before the first scan, taken as the distance between the medial malleolus and the tibial end plate. The leg of each participant will be fitted into a carbon fiber shell and immobilized within the gantry of the scanner for the duration of the scan. A reference line will be placed at the tibial end plate, with the first computed tomography slice taken at 4% and 30% of the tibial length from the reference line for the metaphyseal and diaphyseal tibia. For follow-up measurements at the 4% site, automatic algorithms will match the volumes of interest to baseline scans, using the cross-sectional area (CSA) within the periosteal boundary, so only the bone volume common to the baseline scans will be assessed [48]. The matching algorithms will be disabled for analysis at the 30% site [49]. Daily quality control scans will be performed using a phantom containing hydroxyapatite (HA) rods. The quality of each high-resolution peripheral quantitative computed tomography scan will be reviewed according to the manufacturer’s visual grading system, and poor-quality scans will be excluded. Data processing will be performed as per Boutroy et al [48] and Burghardt et al [50]. The manufacturer’s standard evaluation procedure will be used to derive the following outcomes: total vBMD (mg HA∙cm^3^), trabecular vBMD (mg HA∙cm^3^), cortical vBMD (mg HA∙cm^3^), trabecular bone volume fraction (%), trabecular area (mm^2^), cortical area (mm^2^), cortical thickness (mm), trabecular thickness (mm), trabecular number (mm^−1^), trabecular separation (mm), cortical porosity (%), and cortical pore diameter (mm). The biomechanical properties under uniaxial compression, specifically stiffness (kN/mm) and failure load (kN), will be determined by micro–finite element analysis [51]. Evaluations will be performed by the same researcher to ensure consistency of periosteal and endosteal contouring. The coefficient of variation at the 4% site is 0.2% for total vBMD, 0.4% for trabecular vBMD, 0.9% for cortical vBMD, ≤1.3% for geometry, ≤2.1% for trabecular microarchitecture, 7.8% for cortical porosity, and ≤3.2% for stiffness and failure load [52]. The coefficient of variation at the 30% site is 0.3% for total vBMD, 0.2% for cortical vBMD, ≤0.8% for geometry, 4.9% for cortical porosity, and ≤0.7% for stiffness and failure load [52].

#### Patellar Tendon Properties

##### Overview

Measures of patellar tendon biomechanical properties will be obtained in vivo during voluntary isometric ramped contractions [53] at 6, 12, and 24 weeks post partum. All measurements on the dominant limb will be performed by the same researcher.

##### Tendon Elongation

Patellar tendon elongation will be assessed using real-time B-mode ultrasonography during ramped isometric contractions performed at a 90° knee angle on an isokinetic dynamometer (Biodex Isokinetic Dynamometer, System 4). The participants’ dominant leg will be strapped at the ankle to the knee extension and flexion attachment. Straps will be tightly positioned at the shoulder and hip and across the thigh of the leg being measured. Isometric ramped contractions will be performed over 5 seconds. Participants will gradually increase the push against the dynamometer, aiming to reach maximum force within 5 seconds. Each participant will perform 4 preconditioning contractions to ensure reproducibility of the measurements [53] before completing 3 trial attempts. An ecoabsorptive external marker will be fixed to the skin using surgical tape. The ultrasound transducer will be positioned over the patellar tendon and the external marker. Tendon displacement will be measured as the distance between the line cast by the external marker and the patellar apex. Trials will not be analyzed if the line cast by the external marker moves on the ultrasound image. A calibrated goniometer will be attached to the lateral side of the tested knee to prevent an overestimation of tendon elongation due to tibial translation.

##### Surface Electromyography

Surface electromyography (EMG) will be used to measure muscle activity (DataLOG system, MWX8, Biometrics) from the long head of the *m. biceps femoris*. An EMG sensor (SX230, Biometrics) with 20-mm contact sensor spacing will be applied at a site corresponding to the distal one-third of the length of the muscle [53]. The location of the electrode will be traced onto an acetate sheet to ensure its accurate placement for subsequent tests. The raw EMG signal will be preamplified and filtered using high- and low-pass cutoff filters set at 10 and 500 Hz. The root-mean-square EMG activity of the biceps femoris will be measured during ramped contractions to assess the antagonistic coactivation level of the knee flexors. A maximal knee flexion isometric contraction will be performed to determine biceps femoris maximal activation when acting as an agonist. The maximal activation of the bicep femoris will be measured over a 50-millisecond window at the point of the maximum torque. The antagonistic torque of the knee flexors during a knee extension contraction will be calculated assuming a linear relationship between EMG and the torque [54] to calculate the true knee extensor torque.

##### Patellar Tendon Length and CSA

Resting patellar tendon length and patellar tendon CSA will be measured using real-time B-mode ultrasonography at a fixed 90° knee angle. The distance between the apex of the patella and the tibial tuberosity, recorded using sagittal ultrasound images, will be taken as resting patellar tendon length. Patellar tendon CSA will be measured using the ultrasound probe placed in the transverse plane, and images will be captured at 25%, 50%, and 75% of tendon length. Images will be analyzed offline using ImageJ (v1.50c; National Institute of Health), 3 images will be averaged at each site, and the mean will be used for calculating tendon stress.

##### Patellar Tendon Stiffness and Young Modulus

Patellar tendon force will be calculated by dividing the true knee extensor torque by the estimated patellar tendon moment arm [55]. Patellar tendon stress will be calculated by dividing the tendon force by the tendon CSA. Tendon strain will be calculated as the ratio (%) of tendon displacement to the initial resting tendon length. Force-elongation data will be fitted with a second-order polynomial curve, allowing the assessment of patellar tendon stiffness. The Young modulus will be calculated as tendon stiffness multiplied by the ratio of tendon length to tendon CSA.

#### Muscle Architecture

The muscle architecture of the *m. vastus lateralis* will be assessed at 6, 12, and 24 weeks post partum by the acquisition and analysis of B-mode ultrasonography (MyLab Omega, Esaote) images with a 4- to 15-MHz linear array probe, with the participant lying supine. Images of the dominant leg will be taken at 50% of the length of the and at the midsagittal line of the *m. vastus lateralis*. The scan site will be marked and traced onto acetate with reference points for subsequent tests. The transducer will be aligned to the fascicle plane to allow optimal capture of the fascicles [56]. Muscle thickness, fascicle length, and pennation angle will be assessed using digitizing software (ImageJ v1.50c). Muscle thickness will be measured as the distance between the superficial and deep aponeuroses of the *m. vastus lateralis*. Muscle thickness values will be calculated as the mean of measurements taken at the proximal, central, and distal locations on the captured ultrasound image. The visible portion of the fascicle within the scan window will be measured. The nonvisible portion of the fascicle will be estimated by linear extrapolation of the fascicles and aponeuroses. The linear extrapolation method is a valid technique for measuring vastus lateralis fascicle length [57], and to reduce fascicle extrapolation error (reported as approximately 4% [58]), an average of 3 fascicles across the image will be taken. The pennation angle will be determined as the angle created from the visible insertion of the fascicle into the deep aponeurosis. An average of 3 pennation angles will be taken across the image.

#### Muscle Protein and Collagen Turnover

##### Overview

**Figure 4:**
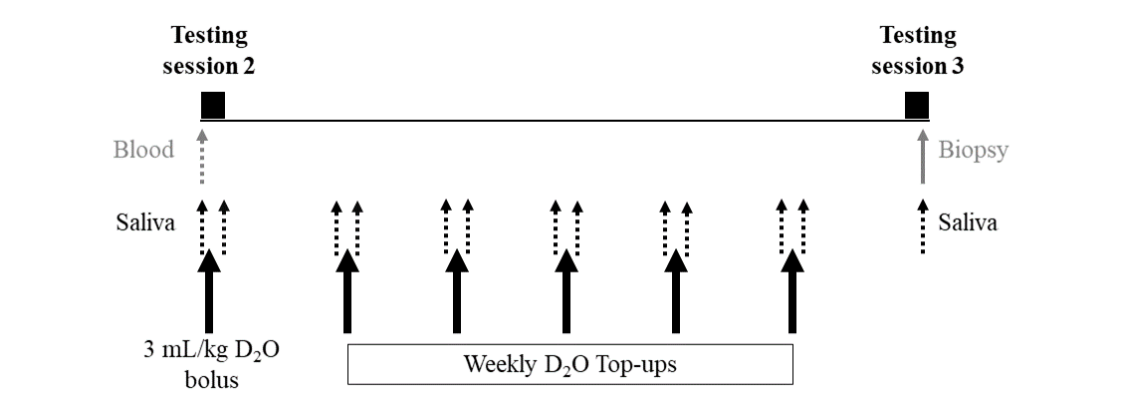
Procedures for muscle protein and collagen turnover using deuterium oxide (D_2_O).

Muscle protein and collagen turnover will be measured between 12 and 18 weeks post partum (Figure 4). Baseline blood and saliva samples will be collected at testing session 2. Participants will then be given a labeled stable isotope tracer (deuterium oxide [D_2_O]) at a dose of 3 mL/kg body weight, divided into 5 smaller doses, and given over 1 hour immediately after the baseline blood and saliva samples are collected. A saliva sample will also be collected 2 to 3 hours after D_2_O ingestion. At testing session 3, a saliva sample and a muscle microbiopsy will be collected.

The blood sample and muscle biopsy will be collected 6 weeks apart. During this 6-week period, participants will take a weekly *top-up* of D_2_O (approximately one-third of the original bolus) at home and will be asked to provide a saliva sample immediately before and 2 to 3 hours after each dose. Because participants will be D_2_O naïve, alanine labeling in plasma proteins reflects the baseline enrichment of body proteins and will be used as a proxy for baseline muscle enrichment. The body water pool will be enriched with D_2_O, and the background enrichment measured from saliva will be compared with the enrichment measured in muscle.

##### Blood Samples

Venous blood will be collected in lithium heparin vacutainers and thereafter centrifuged at 1843 × g and 4 °C for 20 minutes. Plasma will be separated and stored at −80 °C until analysis.

##### Saliva Samples

Saliva will be centrifuged at 4472 × g at 4 °C for 10 minutes to pellet cell debris. The supernatant will be transferred into a clean vial and capped to be stored at −20 °C until analysis.

##### Skeletal Muscle Biopsy

A rested muscle microbiopsy will be collected (Biofeather 14G needle with coaxial cannula [Medax]) from the *m. vastus lateralis* under local anesthetic. Muscle tissue will be immediately stored in dry ice and transferred to a −80 °C freezer for storage pending analysis.

##### Determination of Deuterium Body Water Enrichment

The protocol has been previously described [59,60]; brieﬂy, 100 μL of saliva will be heated in inverted 2-mL vials for 4 hours at 95 °C to purify fractions of body water. The vials will then be cooled on ice and the condensed body water transferred to a clean vial ready for injection on a high-temperature conversion elemental analyzer (Thermo Finnigan, Thermo Scientiﬁc) connected to an isotope ratio mass spectrometer (Delta V advantage, Thermo Scientiﬁc).

##### Determination of Protein-Bound Alanine Enrichment

To assess protein-bound alanine muscle fraction enrichment, approximately 30 mg of muscle will be homogenized in ice-cold homogenization buffer to isolate myoﬁbrillar proteins. After 10 minutes of mixing, samples will be centrifuged at 11,000 × g for 15 minutes at 4 °C. The supernatant (sarcoplasmic fraction) will be separated, and the pellet will be resuspended in 500 μL of mitochondrial extraction buffer. The pellet will then be homogenized using a Dounce homogenizer and centrifuged at 1000 × g for 5 minutes at 4 °C. Insoluble collagen will be separated following centrifugation from myoﬁbrillar proteins, which will be solubilized in 750 μL NaOH, precipitated using 1 M perchloric acid, and pelleted by centrifugation.

The plasma will be deproteinized using ice-cold ethanol (100%) and centrifuged at 17,000 × g for 10 minutes. The myofibrillar, collagen, and plasma pellets will be transferred to boiling tubes containing Dowex with 0.1 M HCl and hydrolyzed overnight at 110 °C for 16 to 20 hours. Following overnight hydrolysis, amino acids will be eluted with 2 M NH_4_OH and dried.

Dried samples will be resuspended in 60 μL of distilled water, 32 μL of methanol, 10 μL of pyridine, and 8 μL of methyl chloroformate with intermittent vortexing. The *n*-methoxycarbonyl methyl esters of the amino acids will then be extracted after adding 100 μL of chloroform. A molecular sieve will be added to remove water for approximately 20 seconds before being transferred to vials. Incorporation of deuterium into the protein-bound alanine will be determined by gas chromatography–pyrolysis–isotope ratio mass spectrometry (Delta V Advantage, Thermo).

##### Calculation of Fractional Synthetic Rate

The fractional synthetic rate (FSR) will be calculated using the incorporation of deuterium in alanine in myofibrillar, collagen, and plasma proteins (APE_Alanine_) and deuterium enrichment of body water from saliva, representing the precursor labeling between the blood sample and the muscle biopsy (APE_Precursor_), corrected for the mean number of deuterium moieties incorporated per alanine, 3.7, and the dilution from the total number of hydrogens in the derivative (ie, 11). The following calculation (Equation 1) will be used, where *t* represents the time between the baseline blood sample and the muscle biopsy:



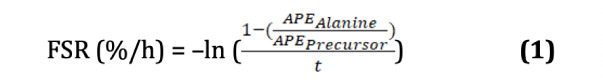



##### Muscle Mass Analyses Using D3-Creatine

**Figure 5:**
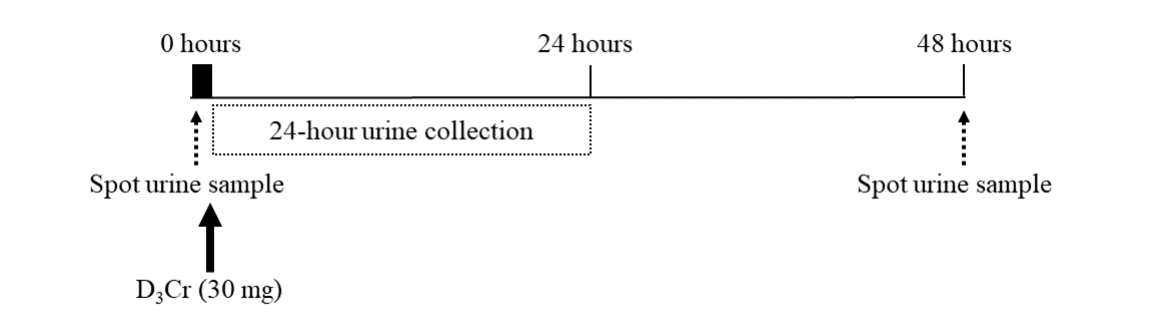
Procedures for muscle mass analyses using D_3_-creatine (D_3_Cr).

Whole-body muscle mass will be estimated using urinary D_3_-creatine at weeks 6, 12, 18, and 24 post partum (Figure 5). A single urine sample will be collected before ingestion of approximately 30 (SD 0.1) mg of D_3_-creatine tracer (D_3_-creatine, CK Isotopes) dissolved in approximately 50 mL of water. Participants will then collect all urine samples for 24 hours, starting immediately after tracer ingestion. A single urine sample will be collected 48 hours after tracer ingestion. The volume of urine collected over 24 hours will be recorded. Approximately 3 mL of each urine sample will be transferred to Eppendorf tubes and stored at −80 °C for later analysis.

Urine will be thawed at room temperature. A standard curve using ^12^C- and ^13^C-creatine will be prepared for the determination of creatine concentration, and a D_3_-creatinine enrichment curve of 0% to 0.1% will be prepared. Once thawed, urine will be mixed, and 10 µL of ^13^C-creatine will be added to 50 µL of urine as an internal standard. A total of 250 µL of ice-cold acetonitrile will be added to samples and standards, vortex mixed, and left to incubate on ice for 30 minutes. Following incubation, samples will be centrifuged at 17,000 × g for 20 minutes. The supernatant will be transferred to vials ready for analysis using high-performance liquid chromatography–mass spectrometry.

From the measurement of urinary D_3_-creatinine enrichment it is possible to calculate total creatine pool size and, therefore, total muscle mass (Equation 2) [61], where *MW*_unlabeled_ and *MW*_labeled_ represent the molecular weights of both unlabeled and labeled creatine, respectively. The estimated creatine pool size will be divided by 4.3 g/kg, reflecting the concentration of creatine found in whole wet muscle mass.



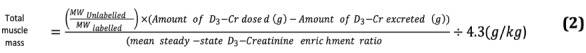



##### Muscle Protein Breakdown Analyses Using D3-3-Methyl-Histidine

**Figure 6:**
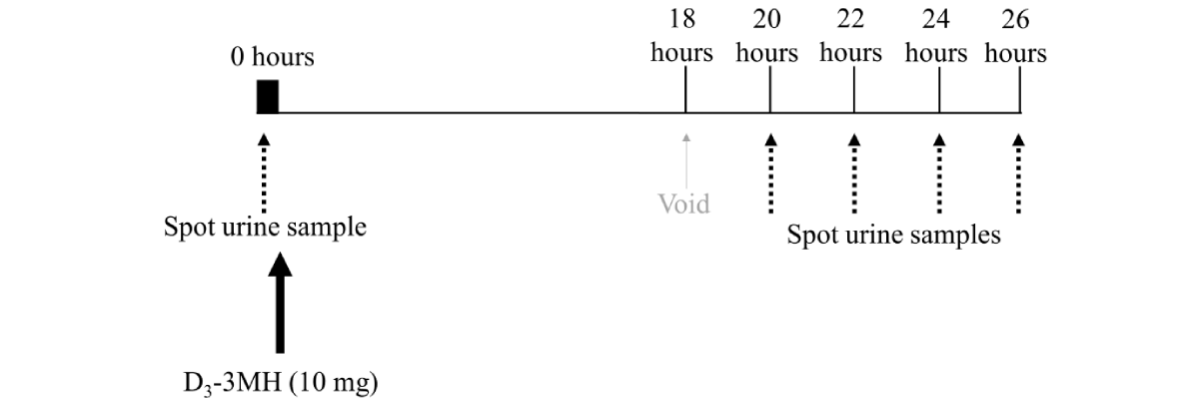
Procedures for muscle protein breakdown using D_3_-3-methyl-histidine (D_3_-3MH).

Muscle protein breakdown will be estimated using D_3_-3-methyl-histidine (D_3_-3MH) measured in urine at weeks 12 and 18 post partum (Figure 6). A single urine sample will be collected before ingestion of 10 mg of a methyl-D_3_-3MH tracer (D_3_-3MH, CK Isotopes) dissolved in 50 mL of water. Participants will void urine 18 hours after tracer ingestion before collecting spot samples at 20, 22, 24, and 26 hours after tracer ingestion. Approximately 3 mL of each urine sample will be stored in Eppendorf tubes at −80 °C until analysis.

A 0% to 10% D_3_-3MH enrichment curve will be prepared as a serial dilution and run alongside the samples. In total, 100 µL of urine will be aliquoted and deproteinized using 1 mL of MeCN:MeOH (1:1). Samples will be vortex mixed and incubated at −20 °C for 1 hour. Samples will be centrifuged at 17,000 × g for 5 minutes at 4 °C. The supernatant will be dried in the Techne Block at <40 °C using nitrogen gas, resuspended using 100 µL of acetonitrile: doubly deionized H_2_O (50:50), and analyzed using high-performance liquid chromatography–mass spectrometry.

The ratio of D_3_-3MH to 3-methyl-histidine will be determined, ratios will be corrected relative to the enrichment curve of each batch, and the atom percent excess will be determined. The predose, baseline urine sample will be used to correct for background enrichment and calculation of the atom percent excess. The ratios will be log-transformed and plotted against time to determine the decay rate.

### Power Calculation

Kraemer et al [62] identified that single-lift strength was 39 (SD 7) kg and 37 (SD 5) kg for women at the end of a 6-month total strength and power and total strength and hypertrophy resistance training protocol, respectively, compared with 32 (SD 4) kg for women undertaking normal field-based military training. Based on the effect sizes between these training and control groups (total strength and power vs control, Cohen *d*=1.23; total strength and hypertrophy vs control, Cohen *d*=1.10), it is anticipated that between 9 and 11 women would be required per group to identify greater posttraining strength in the intervention group than in the control group, with a 1 − β of .80 and α of .05. Hendrickson et al [63] identified that squat and bench press 1 RM were 77 (SD 3) kg and 41 (SD 2) kg, respectively, for women at the end of a 12-week endurance and strength training program, compared with 68 (SD 4) kg and 34 (SD 2) kg for women in the control group, respectively. Based on the effect sizes between the training and control groups (squat, Cohen *d*=2.55; bench press, Cohen *d*=1.62), it is anticipated that between three and six women would be required per group to identify greater posttraining strength in the intervention group than in the control group, with a 1 − β of .80 and α of .05. Hendrickson et al [63], identified that a 3.2-km run time was 18.5 (SD 1.0) minutes for women at the end of a 12-week combined endurance and strength training program, compared with 22.1 (SD 1.2) minutes for women in the control group. Based on the effect sizes between the training and control groups (Cohen *d*=3.26), it is anticipated that 3 women would be required per group to identify greater aerobic fitness in the intervention group than in the control group, with a 1 − β of .80 and α of .05. As such, 11 participants per group would be sufficient to detect improved strength and aerobic fitness in the intervention group compared with the control group. To allow for dropout and noncompliance, we aim to recruit a minimum of 15 participants per group.

### Statistical Analysis

To maximize statistical power, outcomes will be analyzed using mixed-effects linear models to leverage the collection of data on more than 2 time points and to facilitate the analysis of unbalanced data based on participant attrition. Participants will be included as random effects, with group and time included as fixed effects, to assess within- and between-group differences over time. Sensitivity analyses will be conducted by including baseline values in models or other data relative to potential confounders to account for nonrandom allocation. Linear models will be structured to assess within- and between-group differences over time (eg, the inclusion of resistance and HIIT exercise), establishing the rate of change of intervention and control and determining whether these rates are distinct. The suitability of the models will be established by assessing model residuals and transformations made if residuals are not normally distributed or homoscedastic. Uncertainty in parameter estimates will be quantified using the Satterthwaite approximation for *df* values or through bootstrapping procedures. To facilitate interpretation of the magnitude of change across different outcomes, analyses will be standardized relative to the baseline SD, thus placing outcomes on a unitless scale. Principal component analysis will be conducted on change scores to investigate the clustering of outcomes relative to the magnitude of change.

### Ethical Considerations and Consent

This study received ethical approval from the Ministry of Defence Research Ethics Committee (942/MODREC/18) in April 2019. Participants will be provided with an information sheet at least 24 hours before consenting to participation and given a verbal description of the study. Written informed consent will be obtained at the start of the first testing session. Adverse events and reactions will be reported to the trial committee and considered by a minimum of 3 trial committee members.

### Confidentiality and Data Storage

All data will be handled in accordance with the project’s data management plan and in compliance with the General Data Protection Regulation. All raw data will be pseudonymized during the trial. Anonymized data will be uploaded to the active research data storage system at Nottingham Trent University for a minimum of ten years following the completion of the study. Data access rights will be provided only to the relevant members of the research team.

## Results

This study was funded by the Ministry of Defence in March 2018, awarded by BAE Systems (Operations) Limited, contracted through CORDA business (Award Task 0157 proposal reference ASC\CMRCL\RFQ\00694). The study received ethical approval from the Ministry of Defence Research Ethics Committee (942/MODREC/18) in April 2019. Data collection initially started in July 2019, and in March 2020, a total of 4 participants (2 intervention and 2 control) had completed all testing sessions. The study was paused in March 2020 because of the COVID-19 pandemic, but recruitment restarted in May 2021. The study is expected to conclude in September 2022 and the results will be made available thereafter.

## Discussion

### Hypothesis

We hypothesize that occupational physical performance will improve to a greater extent in the training group, who will receive the combined core reconnection and physical development training intervention, than it will in the control group. We also hypothesize that the training group will see greater improvements in all the secondary outcome measures.

### Limitations

Nonrandom allocation could influence participant bias, but it was deemed necessary to use the approach of geographical separation of participants to limit the likelihood of the intervention being shared between the training and control groups. It was deemed unethical for the control group to act as a *true control* (ie, to limit control participants’ physical activity and exercise patterns and levels), and as such, the participants can complete whatever exercise they choose during the study period. Exercise logs will be used to monitor the exercise conducted by the control group, allowing the comparison of any exercise completed between both groups. In addition, it will not be possible for the research team to be blinded to participant group allocation, as the research associates will be involved in both the testing and training of all study participants.

### Dissemination Policy

The trial results will be reported to the Ministry of Defence upon completion of all data collection and analysis. The intent of the Ministry of Defence is to make these core reconnection and physical development training programs available to servicewomen following childbirth under the guidance of their physical training instructors. The evidence provided by this study will inform the management of pregnant and postpartum individuals, building on current Ministry of Defence initiatives such as the provision of female health physiotherapy and bespoke training in pregnancy and postpartum care for physical training instructors. With the approval of the Ministry of Defence, the results will be published in several formats including, but not limited to, peer-reviewed journals, press releases, and conferences.

### Conclusions

Women are now fully integrated throughout the UK Armed Forces, including employment in ground close combat roles. Therefore, high-quality evidence supporting the safe return of women to high levels of occupational fitness following childbirth is required. This study will provide (1) data on the efficacy of a postpartum rehabilitation and physical development program in servicewomen and (2) recommendations for return to occupational fitness following childbirth. In addition, the findings from this study can be used to (1) re-evaluate the current Ministry of Defence postpartum policies and (2) provide education on postpartum requirements, with a view to facilitating the safe and optimal return of postpartum servicewomen to military employment.

## Abbreviations

CSA: cross-sectional area
D_2_O: deuterium oxide
D_3_-3MH: D3-3-methylhistidine
EMG: electromyography
HA: hydroxyapatite
HIIT: high-intensity interval training
HR: heart rate
PFDI-20: Pelvic Floor Distress Inventory–20
POP-Q: Pelvic Organ Prolapse Quantification system
RM: repetition maximum
RPE: rating of perceived exertion
vBMD: volumetric bone mineral density
